# Phasor and neural network approaches for rapid fluorophore fraction analysis in temporal–spectral multiplexed data

**DOI:** 10.1117/1.JBO.30.9.095001

**Published:** 2025-09-08

**Authors:** Jonas Rottmann, Alexander Netaev, Nicolas Schierbaum, Manuel Ligges, Karsten Seidl

**Affiliations:** a Fraunhofer Institute for Microelectronic Circuits and Systems IMS, Duisburg, Germany; b University of Duisburg-Essen, Department of Electronic Components and Circuits, Duisburg, Germany

**Keywords:** time-resolved fluorescence spectroscopy, fluorescence lifetime, phasor analysis, neural networks

## Abstract

**Significance:**

The spatial and temporal distribution of fluorophore fractions in biological and environmental systems contains valuable information about the interactions and dynamics of these systems. To access this information, fluorophore fractions are commonly determined by means of their fluorescence emission spectrum (ES) or lifetime (LT). Combining both dimensions in temporal–spectral multiplexed data enables more accurate fraction determination while requiring advanced and fast analysis methods to handle the increased data complexity and size.

**Aim:**

We introduce two methods, a phasor and a feedforward neural network (FNN) analysis, to extract fluorophore fractions from temporal–spectral data. These methods aim to handle the increased data complexity and size of temporal–spectral multiplexed data and therefore enable access to a more accurate and fast fraction determination.

**Approach:**

The phasor analysis determines the fraction in each dimension and combines them, whereas the FNN is trained using artificially mixed data. Both methods are compared with the reference method using linear combination-based curve fitting (FIT). The methods are tested in a two-component scenario of exogenous fluorophores with different ES and LT and in a three-component scenario of endogenous fluorophores with similar ES and different LT.

**Results:**

In this case, the phasor analysis showed the lowest absolute errors in the fraction determination (1.4% two-component, 4.7% three-component), outperforming the FNN (6.3%) and FIT (8.7%) analysis, which are both not able to recognize all fluorophores in the three-component scenario. The computational effort was reduced by roughly a factor of 6 (Phasor/FNN) compared with FIT.

**Conclusions:**

Both methods demonstrate substantial advantages over common fitting, offering a faster and more accurate determination of fluorophore fractions. These advancements make temporal–spectral multiplexed data more accessible and practical, particularly for high-speed applications.

## 1 Introduction

In fluorescence-based methods such as fluorescence microscopy,^1^ spectroscopy,^2^ and flow cytometry,^3^ the quantification of fluorophore fractions is a key process for molecular characterization and analysis. The fraction information enables insights into cellular structures and molecular dynamics and activities^3^^,^^4^ in biological applications and is also used in material and environmental sciences to study molecular interactions.^2^ Depending on the application, samples are either labeled with exogenous fluorophores or analyzed based on their intrinsic autofluorescence. The fluorescence is typically distinguished by the ES or LT. Although exogenous fluorophores can be designed to differ to a certain degree in ES and LT, autofluorescence samples have intrinsic spectral and temporal characteristics and therefore fixed ES and LT. This inevitably results in spectral overlap^5^ and similar LT,^4^ making it challenging to accurately separate individual fluorophore fractions in fluorophore mixtures.

A multiplexed approach acquires fluorescence decays across different wavelengths λ, combining ES and LT information. Consequently, both information can be analyzed dynamically, enhancing the distinction of fluorophores, even in cases of spectral overlap or similar LT.^6^ In this way, the approach particularly exploits the potential of autofluorescence, eliminating the need for labeling^6^ and enabling applications where labeling is not feasible. The implementation of the approach was shown by Adams et al.,^7^^,^^8^ where the fluorophores were investigated with an algorithmic approach regarding their ES and LT. A mono and double exponential fit was used to identify the containing LT and unmix the fluorophores, although without explicitly quantifying their individual fractions. This demonstrates the capability of the multiplexed approach to analyze fluorophore mixtures and in principle, determining their fraction.

In the analysis of the ES and LT of fluorophore mixtures, common methods, such as fitting, require high computational effort and are vulnerable to noise and dependent on the model assumptions^9^^,^^10^. This limits the accuracy and the analysis time and therefore the application speed, for example, frames per second, in microscopic applications^11^ and the flow rate in flow cytometry.^12^^,^^13^ More advanced and faster methods include phasor^14^ or feed-forward neural network (FNN) analysis.^11^ For phasor analysis, the fluorescence signal is reduced to phasor coordinates by a single-frequency Fourier component analysis, representing the fluorescence characteristic. In contrast to fitting, the phasor transformation is done by performing only two basic computations, making it computationally efficient.^11^ In addition, averaging the decay over the entire signal filters high-frequency signal components, the same as the Fourier analysis of a single frequency. This makes the phasor analysis more robust to noise compared with fitting procedures,^15^ which occurs predominantly at high frequencies. In this way, phasor analysis is able to unmix fluorophores with multi-exponential decays, where fitting is limited due to the needed data quality.^10^ On the other side, due to the reduction of the ES and LT to the phasor coordinates, details of the fluorescence are neglected. Therefore, the phasor analysis is suited and well known for clustering of fluorescence populations^16^ and the determination of fractional contribution,^14^^,^^17^ applied in spectral^17^ and temporal^14^^,^^16^ dimensions. Although the fraction is defined as the ratio of the fluorophore concentration to the overall concentration of all fluorophores, the fractional contribution is defined as the ratio of the signal of the fluorophore to the overall signal.^17^ For FNN analysis, FNNs are trained to recognize the characteristic spectral (ES) or temporal (LT) signatures of the different fluorophores. Due to the absence of iterative calculation cycles in FNN fluorophore analysis, they are also computationally efficient.^11^ FNNs are most commonly applied to distinguish fluorophore components based on either their temporal^5^ or spectral^18^ signatures. Artificial intelligence approaches that exploit both dimensions simultaneously^19^ are also being pursued. Another approach, analyzing only the LT, is the unmixing of fractional contributions in the frequency domain.^10^

To utilize the potential of temporal–spectral data for a precise and rapid determination of the fluorophore fractions, we present and evaluate enhanced phasor-based and FNN analysis methods. The fit-free methods are focused on applications with known input fluorophores and evaluated for the analysis of the data presented by Adams et al.^7^^,^^8^ For the phasor analysis, the fractions are separately determined in spectral and temporal dimensions and afterward combined by weighting based on the distinguishability of the fluorophores and the measurement precision in that dimension. For the FNN analysis, we test the ability of FNNs to recognize the best combination of both dimensions for an accurate determination of the fractions. Furthermore, FNNs are often trained with measured data, resulting in significant experimental effort as a high amount of training samples is required.^5^ In this work, a low-effort method for a fast implementation is tested, where the FNN is trained with artificially mixed data samples, which are created from individual measurements of the fluorophores.

Both methods are tested in a two-component scenario with exogenous fluorophores and a three-component scenario with endogenous fluorophores. Exogenous fluorophores are synthetically manufactured dyes, which are designed to differ in ES and/or LT and are often used as markers in fluorescent applications.^20^ Endogenous fluorophores are molecules within cells and tissues that exhibit autofluorescence and are utilized as natural fluorescent markers in biological analysis.^4^^,^^20^ The exogenous fluorophores in the two-component scenario differ in both, ES and LT, whereas the endogenous fluorophores in the three-component scenario only differ in LT while having a comparable ES. The scenarios enable a comprehensive evaluation of the methods, from a basic two-component scenario differing in both dimensions to a more challenging three-component one varying in LT only. This work demonstrates how these methods enable rapid and precise fraction determination, thereby advancing fluorescence-based analytical applications in biology, material science, and environmental studies.

## 2 Materials and Methods

### 2.1 External Data

The data analyzed in this work were generated by Adams et al.^7^^,^^8^ and collected with a time-resolved spectrometer. The setup records the fluorescence decays over 512 spectral channels from 474.5 to 735.1 nm, resulting in a spectral resolution of 0.51 nm. The temporal resolution is 50 ps, and all samples were measured with three repetitive measurements.

**Fig. 1:**
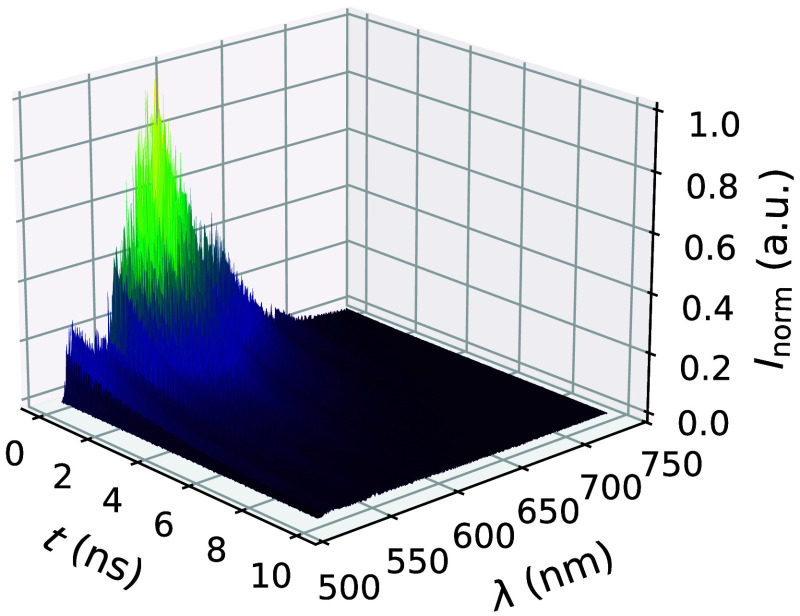
Measured and preprocessed temporal–spectral intensity $I_{\text{norm}}(\lambda ,t)$ of rhodamine B in methanol.

To focus on the fluorescence information, the recorded data are preprocessed. To this end, the first 72 spectral channels are neglected due to a lack of signal because of a dichoridic mirror blocking the excitation laser. Furthermore, only 10 ns of each spectral channel is evaluated, starting from the intensity maximum, and the background level is corrected. The resulting temporal–spectral multiplexed data are exemplary shown in Fig. 1.

The exogenous fluorophores, rhodamine B and fluorescein sodium, differ in ES and LT and were measured by Adams et al.^7^ both, independently and as mixtures. For the independent measurements, rhodamine B was diluted in double-distilled water (RhB-W) and in methanol (RhB-M), whereas fluorescein sodium (FLU) was diluted in phosphate-buffered saline (PBS). Furthermore, two mixtures of FLU in PBS and RhB-M, as well as RhB-W, were measured with the concentrations displayed in Table 1.

**Table 1:** Composition of the exogenous fluorophore mixtures of fluorescein sodium (FLU) and rhodamine B diluted in methanol (RhB-M) and water (RhB-W).

	FLU	RhB-M	RhB-W
EXO-1	0.5 μM (9.1%)	5 μM (90.9%)	—
EXO-2	0.5 μM (9.1%)	—	5 μM (90.9%)

The analyzed endogenous fluorophores in this work are elastin (ELA), flavin adenine dinucleotide (FAD), and riboflavin (RF), which have a similar ES^21^ and differ in LT.^22^ All three fluorophores were diluted in double-distilled water. As the exogenous fluorophores, they were measured independently and as mixtures by Adams et al.^8^ The mixture compositions are listed in Table 2.

**Table 2:** Composition of the endogenous fluorophore mixtures of elastin (ELA), flavin adenine dinucleotide (FAD), and riboflavin (RF).

	ELA	FAD	RF
ENDO-1	500 μM (76.9%)	100 μM (15.4%)	50 μM (7.7%)
ENDO-2	500 μM (83.3%)	50 μM (8.3%)	50 μM (8.3%)
ENDO-3	400 μM (66.7%)	100 μM (16.7%)	100 μM (16.7%)

### 2.2 Artificial Mixing

Besides the measured mixtures, which are used for testing the methods, artificially mixed data are generated in this work to train and validate the FNN. Therefore, a linear superposition of the temporal–spectral fluorescence intensity $I_{\mathrm{mix}}(\lambda ,t)$ is assumed to generate artificial mixtures. The artificial mixtures of different fractions are generated from the individually measured fluorophores with the temporal–spectral-fluorescence intensity $I_{n}(\lambda ,t)$ of j fluorophores and their fractions $f_{n}$

$$I_{\mathrm{mix}}(\lambda ,t)=\overset{j}{\underset{n=1}{\sum }}f_{n}\cdot I_{n}(\lambda ,t).$$ (1)

As described above, the fluorophores are individually measured with different concentrations. Because of this and with the fluorescence intensity approximately scaling linearly to the fluorophore concentration for low concentrations,^23^ the spectral–temporal fluorescence is normalized by the concentration of the fluorophore. The assumed superposition is valid if no interaction between the fluorophores takes place when mixed. Possible interactions are dependent on the specific fluorophores. This work focuses on the rapid determination of fractions of unknown mixtures with known constituents. As the assumption of a linear superposition allows for the generation of artificial training data, this approach avoids the necessity for extensive measurements on a variety of mixtures. However, the availability of sufficient amounts of real experimental training data conceptually allows for the determination of fractions in the presence of interactions.

### 2.3 Lifetime and Spectral Phasors

The phasor coordinates, s and g, represent the sine and cosine components of a modulated signal in the frequency domain, respectively. The lifetime phasors are computed by integrating the product of the signal’s time-dependent exponential decay $I_{\mathrm{LT}}(t)$ with sinusoidal functions at the modulation frequency ω with the following equations:^1^

$$s=\frac{\int_{0}^{\infty }I_{\mathrm{LT}}(t)\cdot \sin(\omega t)dt}{\int_{0}^{\infty }I_{\mathrm{LT}}(t)dt},$$ (2)

$$g=\frac{\int_{0}^{\infty }I_{\mathrm{LT}}(t)\cdot \cos(\omega t)dt}{\int_{0}^{\infty }I_{\mathrm{LT}}(t)dt}.$$ (3)

**Fig. 2:**
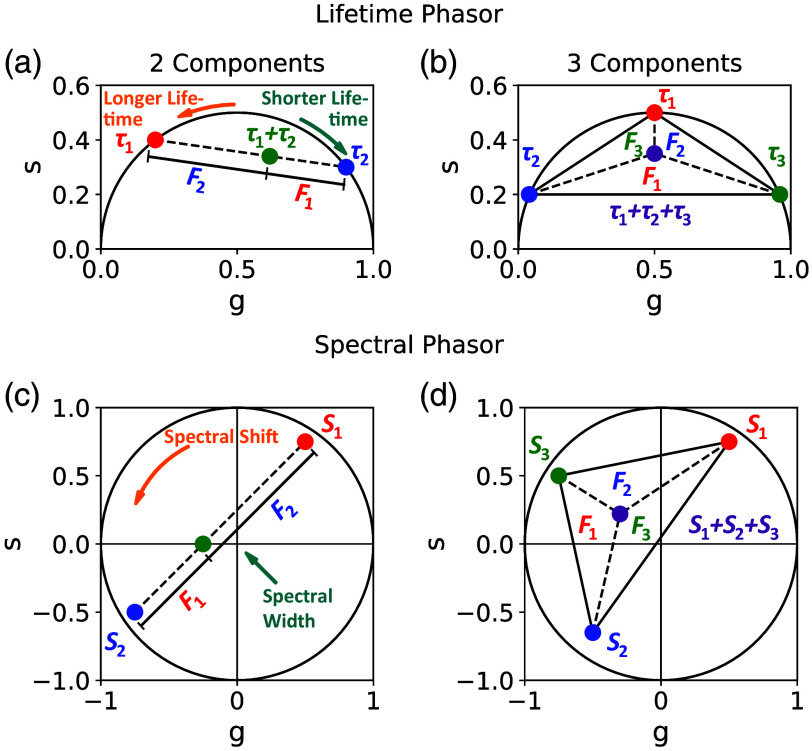
(a) and (b) Phasor lifetime plots with exemplary phasors $\tau_{n}$ of two (a) and three (b) fluorophores and a mixture of these fluorophores. The fractional contribution $F_{n}$ of these fluorophores is calculated by the euclidean distance between the phasors for two fluorophores and the area between the phasors for three fluorophores. (c) and (d) Corresponding phasor plots for spectral phasors $S_{n}$.

For mono exponential decays, the phasors form a semicircle with the LT rising counter-clockwise and multi-exponential decays being inside the semicircle, as seen in Fig. 2(a). Spectral phasors are calculated analog with the modulated signal of the ES $I_{\mathrm{ES}}(\lambda )$ and are displayed in the unit circle, with the angle being dependent on the emission wavelength and the euclidean distance being dependent on the spectral width [Fig. 2(c)]. The modulation frequency ω acts as a scaling factor in phasor analysis and is therefore matched to the LT and ES. In this work, a sine and cosine period of 10 ns for lifetime phasors and 220 nm for spectral phasors is chosen. In addition, phasors of mixtures of two components are located between the individual phasors, whereas phasors of mixtures of three components are inside the triangle of those, depending on their fraction (Fig. 2).^1^^,^^17^

### 2.4 Fractioning with Phasors of Two Fluorophores

Besides clustering of the different fluorophores, phasors can be used to determine the fraction of mixtures. For a mixture of two fluorophores, x and y, the position of the phasor of the mixed one between the individual measurements is depending on their fraction. Accordingly, the euclidean distance between the phasors can be used to determine the fractional contribution $F_{\mathrm{LT}}$ of fluorophore x (Fig. 2).^1^^,^^17^ As mentioned in Sec. 1, the fractional contribution is the part of the signal which one fluorophore contributes to the overall signal, given by the integral of the exponential decay $I_{\mathrm{LT},x}(t)$ with lifetime $\tau_{x}$ and coefficient $A_{\mathrm{LT},x}$ for lifetime phasors

$$F_{\mathrm{LT},x}=\frac{\int_{0}^{\infty }A_{x}\cdot e^{-\frac{t}{\tau_{x}}}dt}{\int_{0}^{\infty }A_{x}\cdot e^{-\frac{t}{\tau_{x}}}dt+\int_{0}^{\infty }A_{y}\cdot e^{-\frac{t}{\tau_{y}}}dt},$$ (4)

$$\text{with }I_{\mathrm{LT},x}(t)=A_{\mathrm{LT},x}\cdot e^{-\frac{t}{\tau_{x}}},$$ (5)

$$\text{and }A_{\mathrm{LT},x}=c_{x}\cdot p_{x}.$$ (6)

The coefficient $A_{\mathrm{LT},x}$ of the decay of fluorophore x is dependent on the fluorophore and setup properties, ranging from the fluorescence yield, concentration $c_{x}$ and excitation and emission spectrum (ES) of the fluorophore to the excitation wavelength and quantum yield of the setup and its detector. The coefficient is proportional to the fluorophore concentration for low concentrations.^23^ Therefore, the coefficient is split up in Eq. (6) into a combination of the concentration $c_{x}$ of the fluorophore and the further properties per concentration $p_{x}$. For sufficiently long measurement times, the integral of the LT is approximately $A_{\mathrm{LT},x}\cdot \tau_{x}$.

This separation of the coefficient in Eq. (6) is necessary to determine the fraction $f_{x}$ of fluorophore x and transform Eq. (4) into Eq. (7) to calculate the ratio of the concentrations of the fluorophores. With the ratio of concentration to overall concentration being defined as a fraction in Sec. 1 and knowing that the mixture consists of only two fluorophores ($f_{x}+f_{y}=1$), the fraction of fluorophore x can be calculated by Eq. (8)

$$\frac{c_{x}}{c_{y}}=\frac{F_{\mathrm{LT},x}}{1-F_{\mathrm{LT},x}}\cdot \frac{p_{y}\cdot \tau_{y}}{p_{x}\cdot \tau_{x}},$$ (7)

$$f_{x}=\frac{1}{1+\frac{c_{y}}{c_{x}}}.$$ (8)

The properties and lifetimes, needed for the calculation of fraction $f_{x}$, are obtained from the data. The properties $p_{x}$ and $p_{y}$ of the fluorophores are calculated as the ratio of the maximum intensity of the exponential decay and the known concentration of the single measurements. In this way, $p_{x}$ and $p_{y}$, as the emission signal properties per concentration, are determined. The lifetime used for the calculation of fractions is calculated by the determined phasors and the modulation frequency ω with^14^

$$\tau =\frac{s}{g}\cdot \frac{1}{\omega }.$$ (9)

The fraction of the second fluorophore $f_{y}$ can be calculated using the same approach or by $f_{y}=1-f_{x}$.

For the spectral phasors, this method can be used in the same way. To this end, the spectral fractional contributions $F_{\mathrm{ES},x}$ can be calculated analog to Eq. (4), with the integral of the spectral intensity $I_{\mathrm{ES},x}$ in the analyzed wavelength interval ($\lambda_{s}$ to $\lambda_{e}$)

$$F_{\mathrm{ES},x}=\frac{\int_{\lambda_{s}}^{\lambda_{e}}I_{\mathrm{ES},x}(\lambda )d\lambda }{\int_{\lambda_{s}}^{\lambda_{e}}I_{\mathrm{ES},x}(\lambda )d\lambda +\int_{\lambda_{s}}^{\lambda_{e}}I_{ES,y}(\lambda )d\lambda }.$$ (10)

### 2.5 Fractioning with Phasors of Three or More Fluorophores

For the calculation of the fraction of mixtures with three fluorophores, x, y, and z, the fractional contributions can be used in a similar way. They are calculated by the areas given by the triangle of the mixture phasor and the phasors of the individual fluorophores, shown in Fig. 2.^1^^,^^17^ The fractional contribution is again given by the ratio of the signal integrals, as seen in Eq. (4), with one more integral being added up for the total signal. This can be resolved to Eqs. (11) and (12) for the ratio of one fluorophore to one of the others and furthermore be used for the calculation of the fraction with Eq. (13)

$$\frac{c_{x}}{c_{y}}=\frac{F_{\mathrm{LT},x}}{1-F_{\mathrm{LT},x}-F_{LT,z}}\cdot \frac{p_{y}\cdot \tau_{y}}{p_{x}\cdot \tau_{x}},$$ (11)

$$\frac{c_{x}}{c_{z}}=\frac{F_{\mathrm{LT},x}}{1-F_{\mathrm{LT},x}-F_{LT,y}}\cdot \frac{p_{z}\cdot \tau_{z}}{p_{x}\cdot \tau_{x}},$$ (12)

$$f_{x}=\frac{1}{1+\frac{c_{y}}{c_{x}}+\frac{c_{z}}{c_{x}}}.$$ (13)

The fractions of the other fluorophores can be calculated the same way, and for the spectral phasors, the fractional contribution is changed to the ES, as previously described.

This approach can also be used to determine the fractions of more than three fluorophores.^11^ Each additional fluorophore adds up a ratio for the concentration of this fluorophore to the others, increasing the complexity of the calculation.

### 2.6 Combination of Lifetime and Spectral Phasors

After determining the fractions with the lifetime (${\mathrm{Ph}}_{\mathrm{LT}}$) and spectral (${\mathrm{Ph}}_{\mathrm{ES}}$) phasor analysis, both results can be combined (${\mathrm{Ph}}_{\mathrm{COM}}$), weighted by the accuracy of the determination in the phasor dimension. The accuracy of the determined fractions of one specific dimension is dependent on the distinguishability of the fluorophores and the precision of the measurement setup in that dimension. The weighted combination enables an accurate determination of the fractions for scenarios with spectral overlap or similar lifetime by prioritizing the dimension with the more accurate results.

In the phasor approach, the distinguishability can be derived from the euclidean distance D of the fluorophore phasors in the phasor plot. For more than two components, the euclidean distances for all fluorophore combination, n=1 to j, are summed. In addition, the precision of the measurement setup can be derived from the sum of standard deviations of the phasor positions STD of the repetitive measurements for fluorophores n to j. The ratio R of both values in Eq. (14) is therefore used to evaluate the accuracy of the fraction in each dimension. With both values being dependent on the modulation frequency ω in the same way, the ratio is independent of ω. For an accurate two-dimensional analysis, the ratios are used to weight the fraction results of temporal (LT) and spectral (ES) dimensions to the combined fraction result $f_{\mathrm{com}}$ in Eq. (15)

$$R=\frac{\sum_{n=1}^{j}D_{n}}{\sum_{n=1}^{j}{\mathrm{STD}}_{n}},$$ (14)

$$f_{\mathrm{com}}=\frac{R_{\mathrm{LT}}}{R_{\mathrm{LT}}+R_{\mathrm{ES}}}\cdot f_{\mathrm{LT}}+\frac{R_{\mathrm{ES}}}{R_{\mathrm{LT}}+R_{\mathrm{ES}}}\cdot f_{\mathrm{ES}}.$$ (15)

### 2.7 Data Preparation and Setup for Phasors

Although the data provide exponential decays over different spectral channels for each sample, the amplitude of each spectral channel is used to define the ES. As the phasor approach is designed to split both dimensions, we prefer this approach over using the LT dependent intensity as the integral of the exponential decay (approximately $A_{\mathrm{LT},x}\cdot \tau_{x}$, as described before), which would couple both dimensions. Furthermore, the ES is normalized and used for the spectral phasor analysis. For the lifetime analysis, each spectral channel provides a lifetime phasor. A threshold is used to exclude phasors of channels with low signals, and the remaining phasors are combined into a weighted mean value for each sample. In this way, the channels with most signal and therefore higher signal to noise ratio are weighted stronger. The fractions are calculated for all combinations of the repetitive fluorophore measurements. The standard deviation Δf of these fractions is used to describe the precision of the results, given by the mean value f.

### 2.8 FNN Setup and Data Preparation

**Fig. 3:**
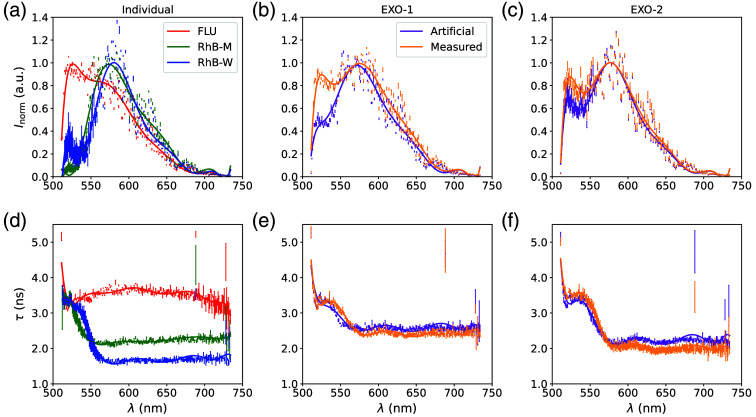
Fluorescence emission spectra and lifetimes of the measurements and artificial mixtures of the exogenous fluorophores: Fluorescein sodium (FLU) and Rhodamine B (RhB) diluted in methanol and water and the mixtures EXO-1 (FLU and RhB in methanol) and EXO-2 (FLU and RhB in water). Panels (a)–(c) show the normalized maximum signal of the spectral channels for the measurements of the exogenous fluorophores and their mixtures, as well as artificial mixtures with identical fractions. Panels (d)–(f) show the corresponding LT of the different wavelength channels determined with a least square mono exponential fit. Lifetime values with standard deviations greater than ±1  ns, due to low intensities, are excluded. The points represent the mean measured values, whereas the line depicts the polynomial fit to the data.^7^

The FNN is set up with the PyTorch library, and the ReLU function is used as the activation function and the Adam optimizer for the loss function. A learning rate of 0.0005 is used, and three hidden layers with decreasing size, by a factor of 10, are implemented. To avoid overfitting and therefore increase the functionality and accuracy of the FNN, the data resolution is reduced.^24^ For this purpose, the signals of two spectral channels are summed, as well as the intensities of two consecutive time bins. This artificially reduces the temporal and spectral resolution by a factor of 2 to a temporal resolution of 100 ps and a spectral resolution of 1.02 nm. This is expected to be still sufficient to resolve lifetimes of several ns and spectral signatures wider than 100 nm (see Figs. 3 and 7). The resulting data with a size of 22000 values is fed into the input layer, leading to hidden layers of size 2220, 222, and 22 and two or three outputs for the fractions of the fluorophores. In addition, each data sample is normalized to determine different fractions independent of the absolute concentration. For a noise-independent norming, the values are normalized with a polynomial fit of the maxima of the channels (see Figs. 3 and 7).

### 2.9 FNN Training and Testing

The FNN is trained with artificial mixtures, created by the individual measurements of the fluorophores with the method presented in Sec. 2.2. The fractions of the artificial mixtures are varied from 0 to 1 in steps of 0.1, and the repetitive measurements of the fluorophores are combined in all possible combinations. For two fluorophores with three repetitive measurements, this equals 192 artificial mixtures, and for three fluorophores with three repetitive measurements, this equals 1782 artificial mixtures available for training.

To validate the FNN and show the accuracy of the method with the given setup, three randomly chosen mixtures are excluded from the created ones and later used to test the trained FNN. The other mixtures (189/1779, respectively) are randomly split into six equal-sized batches, which are used for the training of the network in 1000 epochs. To show the robustness of the method, the FNN is trained and tested 10 times, and the mean fraction results f and the standard deviation Δf are evaluated.

### 2.10 Reference Method and Computational Effort

Fitting a multi-exponential function to the data would provide fractions without prior information and measurements of the individual fluorophores in the phasor and FNN analysis. As described by Kremers et al.,^10^ fitting of multi-exponential functions for unmixing requires high-quality data, which is often not provided by biological samples, such as the endogenous fluorophores used in our work. In addition, it requires a high amount of computational iterations and is therefore not suited as a fast reference method. Because of this, a linear combination of the individual measured components based on curve fitting (FIT) is used as a reference for the developed methods. The linear combination was shown before in Eq. (1), and the curve fitting identifies the fractions $f_{n}$ for the best fit of the measured data. For the analysis of all spectral channels, their decays are transformed into a one-dimensional sequence, and curve fitting is applied. Restrictions for the fitting are the sum of the components is 1 and all components are between 0 and 1. For the evaluation of the method, only the mean fraction results $f_{n}$ of the repetitive measurements are analyzed.

The computational effort of the different methods is determined by the time needed for the calculation. For statistical reasons, the curve fit, phasor calculation, and FNN test are run 100 times, and the mean computational time is calculated. The calculations are run on an i5-8265U (Intel, Santa Clara, United States). Although the analysis is dependent on the used algorithms and hardware in this work, it still provides a valuable estimate of the computational effort and possible application speed.

## 3 Results

### 3.1 Exogenous Fluorophores

For the exogenous fluorophores, the previously described mixtures of FLU, RhB-M, and RhB-W are analyzed (Table 1). The spectral and temporal information of the individual measurements and mixtures are shown in Fig. 3. In doing so, the spectral information refers to the maximum intensity of each channel and is normalized by a polynomial fit. The lifetime is calculated with a mono-exponential fit. The data are processed, as described in Sec. 2.8, and the data refer to the mean values and the error bars to the standard deviation of the repetitive measurements.

**Fig. 4:**
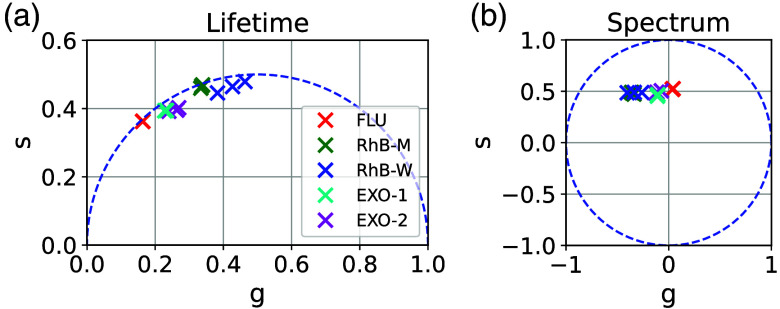
Temporal (a) and spectral (b) phasors for the repetitive measurements of the individual exogenous fluorophores and their mixtures.

The ES of the individual fluorophores in Fig. 3(a) shows a difference in emission wavelength between FLU and RhB, with an emission maximum of FLU at 525 nm. RhB has similar ES in methanol and water, with an emission maximum at 574 nm (methanol) and 581 nm (water), respectively. The difference in emission maximum is based on the different solvents. The results are in accordance with the literature.^25^^,^^26^ This difference is also seen in the spectral phasor plot [Fig. 4(b)]. The ES of the mixtures is shown in Figs. 3(b) and 3(c). In addition to the measured mixture data, the artificial mixture data, which are used for the training of the FNN, are shown. The measured ES of the mixtures shows peaks of both fluorophores for both mixtures. Although the ESs of the artificial and measured mixtures are similar for wavelengths >550  nm, they differ for wavelengths <550  nm. This difference is more distinct for EXO-1, compared with EXO-2. As expected, the mixture phasors are located between the phasors of the individual fluorophores, with higher variance in Rhb-W phasors compared with FLU and RhB-M [Fig. 4(b)].

**Fig. 5:**
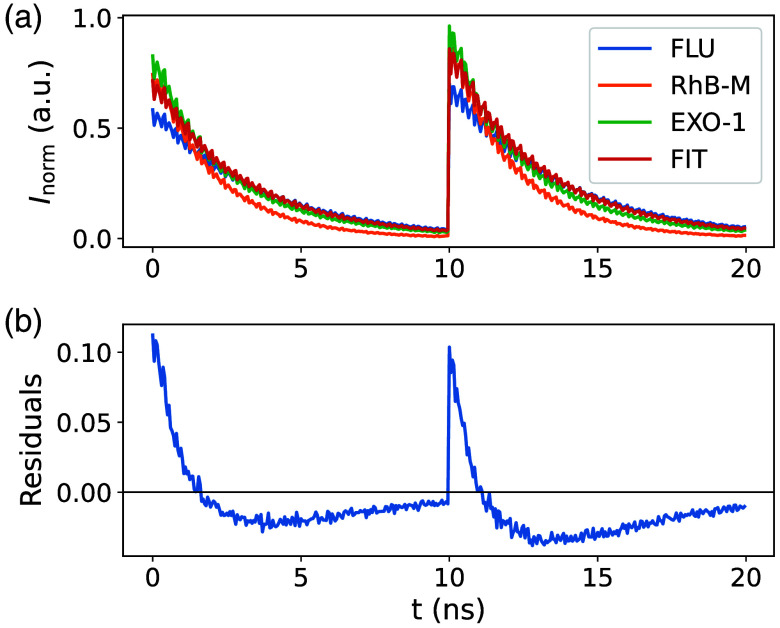
Mean normalized decay of FLU, RhB-M, and EXI-1 and the FIT analysis function for the two exemplary spectral channels from 553.6 to 554.1 nm and from 554.1 to 554.6 nm. The spectral channels are transformed into a one-dimensional sequence as used for the FIT analysis, resulting in the exemplary time trace shown (a), where the first and second 10 ns periods correspond to the first and second spectral channels, respectively. The corresponding fitting residuals are shown in panel (b).

The LTs of the fluorophores are shown in Fig. 3(d) and are constant for wavelength >575  nm. The LT for FLU is around 3.5 ns, for RhB-M 2.2 ns and for RhB-W 1.7 ns, which is in accordance with the literature.^7^ The LT rises for wavelengths <550  nm, which was seen before by Adams et al.^7^ Lifetime phasors are close to the half circle, showing a mono exponential LT and again a higher variance in RhB-W phasors compared with FLU and RhB-M, as well as mixture phasors between the individual ones [Fig. 4(a)]. The LT of the measured and artificial mixed data is depicted in Figs. 3(e) and 3(f), showing a similar behavior with a slightly longer LT of around 0.2 ns of the artificial mixtures for wavelengths >570  nm. The FIT analysis and the residuals are shown exemplarily in Fig. 5(a). The FIT does not exactly match the EXO-1 delay as the beginning of the decay is fitted with a lower intensity and the end with a higher one. This results in the systematic error, which is seen in the residuals [see Fig. 5(b)]. The reduced $\chi^{2}$ equals 60.8 (EXO-1) and 220.1 (EXO-2) for all spectral channels.

**Fig. 6:**
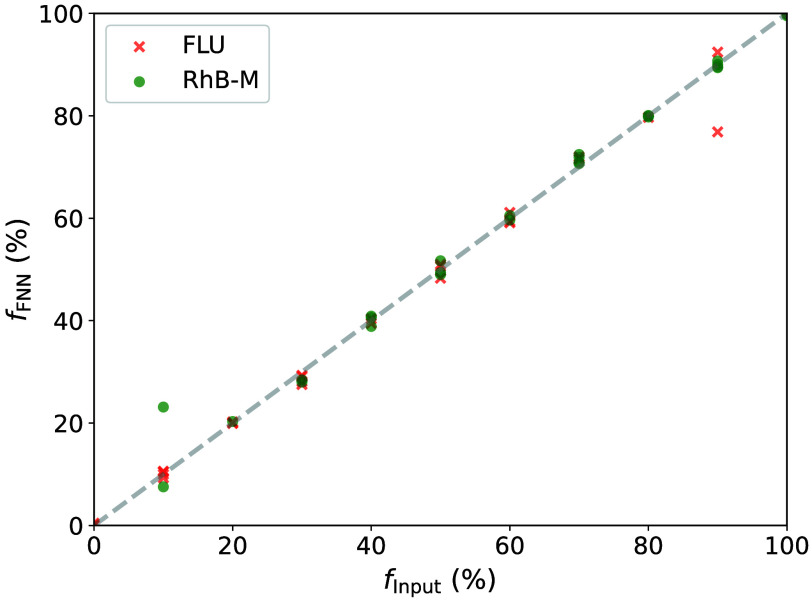
Validation results of the FNN for mixtures of fluorescein sodium (FLU) and rhodamine B in methanol (RhB-M) with the set fractions $f_{\text{Input}}$ and determined fractions $f_{\mathrm{FNN}}$ of the FNN of different mixtures.

The results of the validation of the FNN for artificial FLU-RhB-M mixtures are shown in Fig. 6. As described in Sec. 2.9, they demonstrate the ability and accuracy of the FNN to determine fractions of artificially mixed samples, which were excluded from training. Therefore, they are unknown to the network and correspond to a measured mixture with ideal superposition and no interaction of the different samples. The mean absolute error of the determined fractions $f_{x}$ of the FLU-RhB-M mixtures of the validation is 0.4%, and the standard deviation $\Delta f_{x}$ is ±2.6%. The mean absolute error of the fractions $f_{x}$ of the FLU-RhB-W mixtures of the validation is 0.1% with a standard deviation $\Delta f_{x}$ of ±3.8%.

The determined fractions of the fluorophores with the different methods are shown in Table 3. Due to the differences of the fluorophores in both dimensions, the phasor analysis achieves high accuracy in temporal (1.6% mean absolute error) and spectral (1.2%) dimensions. Furthermore, the differences in LT and ES result in a weighting of 60/40 (lifetime/spectral) for EXO-1 and 55/45 for EXO-2 in the phasor combination. The combined phasor analysis with this weighting achieves a mean absolute error of the determined fractions of 1.4%. The results of the FNN show a significantly higher absolute error of 11.3% for the fraction determination of EXO-1. By contrast, EXO-2 is determined to be more accurate with an absolute error of 1.2%. This results in a mean absolute deviation of 6.3%, with standard deviations $\Delta f_{x}$ comparable to the phasor analysis. The FIT analysis shows a similar result for both exogenous mixtures to the FNN, with an error of 13.0% (EXO-1) and 4.4% (EXO-2), resulting in a mean absolute error of 8.7%. The higher variance in RhB-W data in EXO-2 compared with RhB-M data in EXO-1 results in a higher standard deviation $\Delta f_{x}$ of EXO-2 (1.7% to 2.8%) compared to EXO-1 (0.5% to 1.0%) for all methods.

**Table 3:** Determined fractions $f_{x}$ of the mixtures EXO-1 and EXO-2. The fractions were determined with the spectral (${\mathrm{Ph}}_{\mathrm{ES}}$) and lifetime (${\mathrm{Ph}}_{\mathrm{LT}}$) phasor analysis, as well as the combined phasor analysis (${\mathrm{Ph}}_{\mathrm{COM}}$), the FNN and the FIT. Standard deviations $\Delta f_{x}$ are provided, and all values are given in percent (%).

EXO-1				
9.1% FLU, 90.9% RhB-M				
	$f_{\mathrm{FLU}}$	$f_{\mathrm{RhB}-M}$	$\Delta f_{\mathrm{FLU}}$	$\Delta f_{\mathrm{RhB}-M}$
${\mathrm{Ph}}_{\mathrm{LT}}$	11.2	88.8	0.5	0.5
${\mathrm{Ph}}_{\mathrm{ES}}$	11.4	88.6	0.5	0.5
${\mathrm{Ph}}_{\mathrm{COM}}$	11.3	88.7	0.5	0.5
FNN	20.4	79.6	1.0	1.0
FIT	22.1	77.9	—	—
EXO-2				
9.1% $f_{\mathrm{FLU}}$, 90.9% $f_{\mathrm{RhB}-W}$				
	$f_{\mathrm{FLU}}$	$f_{\mathrm{RhB}-W}$	$\Delta f_{\mathrm{FLU}}$	$\Delta f_{\mathrm{RhB}-W}$
${\mathrm{Ph}}_{\mathrm{LT}}$	8.0	92.0	1.7	1.7
${\mathrm{Ph}}_{\mathrm{ES}}$	9.1	90.9	2.8	2.8
${\mathrm{Ph}}_{\mathrm{COM}}$	8.5	91.5	2.2	2.2
FNN	10.3	89.7	2.5	2.5
FIT	13.5	86.5	—	—

### 3.2 Endogenous Fluorophores

**Fig. 7:**
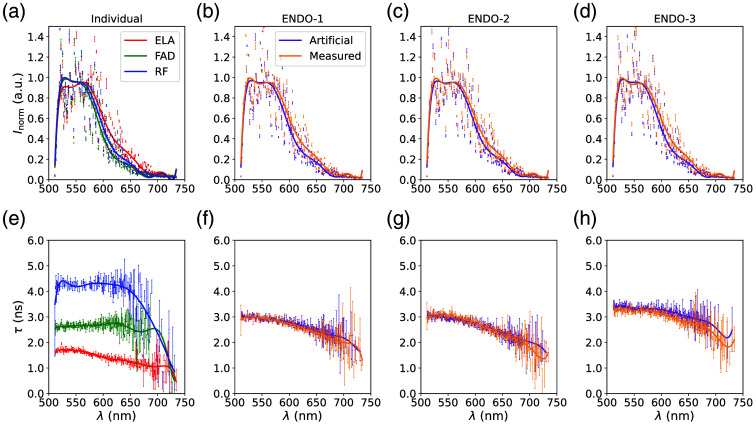
Fluorescence emission spectra and lifetimes of the measurements and artificial mixtures of the endogenous fluorophores: elastin (ELA), flavin adenine dinucleotide (FAD), and riboflavin (RF) and mixtures ENDO-1, ENDO-2, and ENDO-3 of these fluorophores. Panels (a)–(d) show the normalized maximum signal of the spectral channels for the measurements of the endogenous fluorophores and their mixtures, as well as artificial mixtures with identical fractions. Panels (e)–(h) show the corresponding LT of the different wavelength channels determined with a least square mono exponential fit. Lifetime values with standard deviations greater than ±1  ns, due to low intensities, are excluded. The points represent the mean measured values, whereas the line depicts the polynomial fit to the data.^8^

**Fig. 8:**
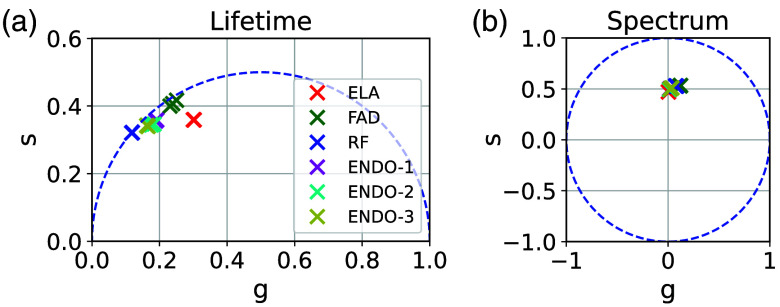
Temporal (a) and spectral (b) phasors for the repetitive measurements of the individual endogenous fluorophores and their mixtures.

The spectral and lifetime information of the endogenous fluorophores and their mixtures are shown in Fig. 7. As expected,^21^ the ESs of the fluorophores are similar. Nevertheless, small differences occur in emission peak (563 nm for ELA, 530 nm for FAD and RF) and in a slightly higher emission for ELA for wavelengths from 600 to 680 nm. Due to the similar ES of the individual fluorophores, the ES of the measured and artificial mixtures looks similar too, with slightly higher intensities of the artificial mixtures for wavelengths between 600 and 680 nm. The phasors of the ESs are shown in Fig. 8(b) and overlap due to the similarities.

In the temporal dimension, LTs of the fluorophores differ and are about 1.9 ns (ELA), 2.7 ns (FAD), and 4.2 ns (RF), which is in accordance with the literature.^22^ High errors in lifetime for wavelength >650  nm occur due to low intensities and can be neglected. The mixtures show different LTs based on their composition, and the LTs are comparable to the corresponding artificially mixed compositions. The lifetime phasors are plotted in Fig. 8(a) and show expected differences. In addition, the position of the ELA phasors inside the circle indicates a multi-exponential decay of ELA.

Furthermore, the residuals of the FIT of the endogenous fluorophores are comparable with the ones of the exogenous fluorophores but with a smaller systematic error. This corresponds to reduced $\chi^{2}$ equal to 53.8 (ENDO-1), 27.8 (ENDO-2), and 62.1 (ENDO-3) for all spectral channels.

The determined fractions of the fluorophores with the different methods are shown in Table 4. Due to the differences in LT and similarities in ES, the phasor lifetime analysis determines the fractions with a mean absolute error of 4.2%, whereas the phasor spectral analysis determines the fractions with a higher mean absolute error of 9.6%. The higher difference in LT of the individual fluorophores leads to a weighting of 85/15 (lifetime/spectral) in the combined phasor analysis, resulting in a mean absolute error of 4.7%. The validation of the FNN for the endogenous fluorophores shows mean errors of <0.4% with standard deviations of <2.3%. In contrast to the high accuracy in validation, the results of the FNN in Table 4 show high absolute errors. All three mixtures are similarly determined by the FNN with high fractions of ELA (>90%), nearly no FAD and small amounts of RF (4% to 6%). This results in a high mean absolute error of 11.8%. Due to the high errors, a further approach focused on the differences in LT and therefore without the spectral information was tested. To neglect the spectral information, all spectral channels are individually normalized to the same intensity of 1, and the later wavelengths from 684.1 to 735.1 nm (100 spectral channels) are excluded due to low intensities. The results of this approach ${\mathrm{FNN}}_{\mathrm{LT}}$, also listed in Table 4, show a high accuracy with a mean absolute error of 1.0%. The FIT shows similar results to the FNN with high determined fractions of ELA (83.8% to 89.9%), nearly no detection of FAD and small fractions of RF (10.1% to 16.2%). This leads to a mean absolute error of 9.1%.

**Table 4:** Determined fractions f of the mixtures ENDO-1, ENDO-2, and ENDO-3. The fractions were determined with the spectral (${\mathrm{Ph}}_{\mathrm{ES}}$) and lifetime (${\mathrm{Ph}}_{\mathrm{LT}}$) phasor analysis, as well as the combined phasor analysis (${\mathrm{Ph}}_{\mathrm{COM}}$), the FNN and the FNN focused on the temporal information only (${\mathrm{FNN}}_{\mathrm{LT}}$) and the FIT analysis. Standard deviations $\Delta f_{x}$ are provided and all values are given in percent (%).

ENDO-1						
76.9% $f_{\mathrm{ELA}}$, 15.4% $f_{\mathrm{FAD}}$, 7.7% $f_{\mathrm{RF}}$						
	$f_{\mathrm{ELA}}$	$f_{\mathrm{FAD}}$	$f_{\mathrm{RF}}$	$\Delta f_{\mathrm{ELA}}$	$\Delta f_{\mathrm{FAD}}$	$\Delta f_{\mathrm{RF}}$
${\mathrm{Ph}}_{\mathrm{LT}}$	66.9	25.6	7.5	2.7	2.8	0.6
${\mathrm{Ph}}_{\mathrm{ES}}$	58.5	23.9	17.6	10.7	7.2	3.5
${\mathrm{Ph}}_{\mathrm{COM}}$	65.6	25.3	9.1	3.9	3.5	1.0
FNN	95.0	0.6	4.5	1.3	0.5	0.9
${\mathrm{FNN}}_{\mathrm{LT}}$	79.1	15.1	5.9	2.2	1.5	1.1
FIT	89.9	0.0	10.1	—	—	—
ENDO-2						
83.3% $f_{\mathrm{ELA}}$, 8.3% $f_{\mathrm{FAD}}$, 8.3% $f_{\mathrm{RF}}$						
	$f_{\mathrm{ELA}}$	$f_{\mathrm{FAD}}$	$f_{\mathrm{RF}}$	$\Delta f_{\mathrm{ELA}}$	$\Delta f_{\mathrm{FAD}}$	$\Delta f_{\mathrm{RF}}$
${\mathrm{Ph}}_{\mathrm{LT}}$	77.4	13.8	8.9	3.0	2.1	1.2
${\mathrm{Ph}}_{\mathrm{ES}}$	66.9	18.2	14.9	10.5	6.7	4.1
${\mathrm{Ph}}_{\mathrm{COM}}$	75.7	14.4	9.8	4.1	2.8	1.6
FNN	93.4	0.6	6.0	1.7	0.7	1.3
${\mathrm{FNN}}_{\mathrm{LT}}$	83.8	8.8	7.4	3.5	1.8	1.8
FIT	88.6	0.0	11.4	—	—	—
ENDO-3						
66.7% $f_{\mathrm{ELA}}$, 16.7% $f_{\mathrm{FAD}}$, 16.7% $f_{\mathrm{RF}}$						
	$f_{\mathrm{ELA}}$	$f_{\mathrm{FAD}}$	$f_{\mathrm{RF}}$	$\Delta f_{\mathrm{ELA}}$	$\Delta f_{\mathrm{FAD}}$	$\Delta f_{\mathrm{RF}}$
${\mathrm{Ph}}_{\mathrm{LT}}$	68.0	18.1	13.9	6.3	4.8	1.7
${\mathrm{Ph}}_{\mathrm{ES}}$	58.2	22.8	19.0	11.8	8.0	4.3
${\mathrm{Ph}}_{\mathrm{COM}}$	66.5	18.8	14.7	7.1	5.3	2.1
FNN	91.6	0.2	8.2	1.9	0.2	1.8
${\mathrm{FNN}}_{\mathrm{LT}}$	65.2	18.2	16.6	3.2	2.1	1.2
FIT	83.8	0.0	16.2	—	—	—

The computational effort is determined as described above for the analysis of the endogenous fluorophores. The time consumption for the calculation of 440 temporal phasors, one for each spectral channel, and 1 spectral phasor equals 9.7 ms and for the calculation of the fractions with a trained FNN 8.7 ms. By contrast, the calculation of the curve fit requires 54.5 ms. This means an exemplary reduction of the computational effort by a factor of 5.6 (Phasor) and 6.3 (FNN) for the computational setup used in this work.

## 4 Discussion

The results demonstrate that all three methods can be used to extract fluorophore fractions from temporal–spectral data, whereas only the phasor method reliably determines fractions in both scenarios. In the two-component scenario, the FNN analysis determines the fractions of EXO-2 with high accuracy (1.2% mean absolute error) while overestimating the fraction of FLU in EXO-1 by 11.3%. Comparing the measured with the artificial mixtures in Fig. 3(b), the difference in the ES between the measured and artificially mixed training data is expected to lead to the increased error in EXO-1. Thereby, the dominant emission peak at 520 nm, corresponding to the emission of FLU, is more distinct in the measured EXO-1 than in the artificial EXO-1. Due to this, the FNN was trained on a lower intensity at the FLU emission peak than observed in the measurements for the given mixture. Consequently, it overestimates the fraction of FLU in EXO-1 by 11.3%. The differences between the measured and artificial data likely arise from interactions between the fluorophores and their solvents, which, as previously described, are not accounted for in the artificial mixing. The different solvents are expected to be the most dominant factor in the data, with FLU being measured in PBS and afterward mixed with RhB in methanol and water. The polarity of the solvent has a strong impact on the fluorescence intensity^27^ and the LT.^28^ In the given scenario the polarity, described by the dielectric constant ε, of water (ε=80)^29^ and PBS (ε=72)^30^ is similar, whereas the polarity of methanol (ε=33)^29^ differs more from the others, increasing the fluorescence intensity^27^ and decreasing the LT^28^ of FLU. Due to this, the superposition of the artificial mixing is more valid for EXO-2 than EXO-1, resulting in higher accuracies for EXO-2.

The FIT analysis shows a similar behavior for EXO-1 in the 2-component scenario as the FNN, by overestimating the fraction of FLU in EXO-1 by 13.0% and 4.4% in EXO-2, resulting in a mean absolute error of 8.7%. The errors likely arise because the curve fitting analyzes simultaneously both, the decay constant (LT) and intensity (ES). As both parameters are optimized together, deviations in one can introduce compensatory errors in the other, leading to inaccurately determined fractions. This is seen in the fitted decay and residuals as the fit compensates for lower intensities at the beginning of the decay with lower intensities at the end of the decay. This systematic deviation is also indicated by high $\chi^{2}$ of 60.8 (EXO-1) and 220.1 (EXO-2). As described before, fitting methods with multi-exponential decays were not applied in this work due to the data quality. A determination of fractional contributions of two fluorophores with a double-exponential fit was demonstrated by Shcheslavskiy et al.,^31^ without an evaluation of the accuracy. Furthermore, their system extends the multiplexed analysis using different excitation wavelengths as another dimension. This dimension could be integrated into the phasor approach with further phasor plots for each excitation wavelength and weighting them as shown before. In the FNN analysis, it could be integrated by adding further input data.

In the three-component scenario, the FNN (11.8% mean absolute error) and FIT (9.1%) analysis do not accurately determine the fractions as the fraction of FAD is determined with less than 0.6% for all mixtures. Therefore, it is not recognized by both methods. However, the validation of the FNN using artificial mixtures shows that the FNN can be used to extract fractions from tempo-spectral data with similar ES. Furthermore, the presence of the fraction information in the data is confirmed by the accurate analysis of the fractions with a mean absolute error of 1.0%, when the similar spectral information is discarded and only the lifetime information is analyzed in ${\mathrm{FNN}}_{\mathrm{LT}}$. Due to this, the FNN is likely to pick up misleading and heavily weighted information from the ES. The ES information is reflected in the intensity of the decay in the different spectral channels. Therefore, it affects each data value within the decay, increasing or decreasing the entire decay curve proportionally to the ES. As a result, changes in the ES are directly reflected in each data value, generating significant differences in the decay curves. By contrast, LT is visible in the data but only indirectly as it affects the rate of the intensity decay over time. Unlike ES, which scales the entire decay curve, LT primarily affects the rate of decay, making its effect more subtle. This is reflected in gradual changes between consecutive intensity values rather than a uniform shift. Consequently, changes in LT result in smaller differences in the decay curves compared with changes in ES. With the given results, it seems that the FNN picks up smaller differences in ES due to their direct appearance better than larger differences in the LT. As previously described, the ES is more intense from 600 to 680 nm for ELA compared with FAD and RF, which is also seen in the measured mixture data compared with the artificial one. This explains the higher estimated fractions of ELA, which is compensated for in the mixed LT by the higher LT of RF compared with FAD. A similar effect is expected for the FIT analysis. As convolutional neural networks (CNNs) are specialized in feature extraction,^32^ a CNN is expected to capture the LT feature better than an FNN. However, tests with a CNN of comparable structure showed no improvement compared with the FNN. By contrast, a deep learning CNN was successfully applied for unmixing of fractional contributions by Smith et al.^19^ Although artificially mixed training data are used in this work, Smith et al. used a set of fully simulated data for training and only 16 spectral channels, compared with 512 in this work.

In contrast to the FNN and FIT analyses, the phasor analysis is designed to analyze the fractions separately in each dimension and combine the results weighted by the distinguishability in the dimension. In the two-component scenario, the distinguishability is given in both, temporal and spectral, dimensions. As a result, the phasor analysis determines the fraction in both dimensions with a low mean absolute error of 1.6% (LT) and 1.2% (ES). As designed in the phasor combination, the differences in both dimensions lead to a balanced weighting of 60/40 (lifetime/spectral) for EXO-1 and 55/45 for EXO-2 and a low mean absolute error in the combined analysis of 1.4%. In the three-component scenario, the differences in LT lead to a low mean absolute error of 4.2% in phasor LT analysis ${\mathrm{Ph}}_{\mathrm{LT}}$, whereas the similar ES results in a higher mean absolute error of 9.6% in phasor ES analysis ${\mathrm{Ph}}_{\mathrm{ES}}$. This is still better than expected from the spectral phasor overlap in Fig. 8(b). Due to the significant differences in LT compared with ES, the combination algorithm is expected to weight the more accurate LT fraction results higher than the ES fraction results. This is confirmed by a weighting of 85/15 (lifetime/spectral) in the three-component scenario, showing that the combination algorithm successfully chooses the dimension of higher distinguishability. In this way, a mean absolute error of 4.7% is achieved by the combined analysis.

The results show that the FNN and FIT struggle with the separation of ES and LT information and the weighting of these dimensions, which is especially seen in the three-component scenario. By contrast, the weighting of the different dimensions was successfully implemented in the phasor analysis, which is responsible for its accurate fraction determination. As a result, the phasor analysis is the only method in this study that consistently provides reliable results across both scenarios and all mixtures. All of the methods presented in this work require information about the mixed fluorophores, given by the individual measurements. An unmixing without this information with a multi-exponential fit is not possible due to the high requirements for data quality, which are not met with the available data.

A comparable FNN approach to determine fluorophore fractions of a two-component and three-component scenario by the LT was presented by Netaev et al.^5^ Using simulated and measured training data, accuracies of 3.0% (two-component) and 5.3% (three-component) were achieved. These accuracies are comparable to the ones achieved by phasor (1.4%; 4.7%) and FNN (6.3%; two-components) analysis. Compared with Netaev et al.,^5^ where fluorophores with different LT are analyzed in the temporal dimension only, the phasor analysis achieves this accuracy for fluorophore with different LT and similar ES. This shows the potential of the phasor analysis, which automatically weights the dimensions and in this way achieves competitive accuracies in temporal–spectral analysis to pre-focused methods. Further comparison is limited due to differences in data quality.

Besides the higher accuracy, phasor and FNN analysis were expected to enable higher application speeds due to simpler calculations. This was proven by a reduction of the computational effort by a factor of 5.6 (Phasor) and 6.3 (FNN).

## 5 Conclusion

In this work, we developed and validated phasor-based and neural network methods for extracting fluorophore fractions from temporal–spectral multiplexed fluorescence data. Both methods were further compared with a conventional linear combination-based curve-fitting method. Although both FNN and FIT analyze the temporal and spectral dimensions simultaneously, they predominantly focus on spectral information due to its strong influence on the decay signal. Because of this, the methods are able to determine fractions in a two-component scenario with differences in LT and ES with an accuracy of 6.3% (FNN) and 8.7% (FIT) while not being able to identify all components of a three-component scenario with differences mainly in LT. By contrast, the phasor approach analyzes the temporal and spectral dimensions separately, weighting them based on fluorophore distinguishability and the precision of the measurement setup in that dimension. This results in the highest accuracies for the two-component (1.4%) and the three-component (4.7%) scenario. In addition, computational effort was reduced by a factor of 5.6 (Phasor) and 6.3 (FNN) compared with FIT. The findings demonstrate that the phasor approach significantly improves the accuracy and the speed of fluorophore fraction determination with temporal–spectral multiplexed fluorescence data, making it highly suitable for precise and high-speed applications. Meanwhile, the FNN approach holds potential for further optimization to improve accuracy in more complex scenarios. Future work should focus on implementing and testing these methods in real-world applications, such as high-throughput diagnostics, environmental monitoring, and material science.

## Data Availability

We used data from the University of Edinburgh.^7^^,^^8^ As stated by Adams et al.,^8^ they are available upon request from the University of Edinburgh. The codes for the Phasor, FNN, and FIT analysis are available upon reasonable request to the corresponding author.
